# Effects of spices mixture and cooking on phytochemical content in Ethiopian spicy hot red pepper products

**DOI:** 10.1002/fsn3.3886

**Published:** 2023-12-07

**Authors:** Tadewos Hadero Medalcho, Kebede Abegaz Ali, Engeda Dessalegn Augchew, Juan Ignacio Mate

**Affiliations:** ^1^ School of Nutrition, Food Science and Technology, College of Agriculture, Hawassa University Hawassa Ethiopia; ^2^ Hawassa College of Teacher Education Hawassa Ethiopia; ^3^ Public University of Navarra (UPNA) Pamplona Navarra Spain

**Keywords:** cooked product or sauté, hot red pepper, phytochemicals, spices, spicy hot red pepper

## Abstract

Spicy hot red pepper, the most popular spice in Ethiopia, is also locally known as *berbere*, which is highly valued for its pungency, flavor, and color. The spicy hot red pepper powder is used to flavor *shiro* and other stews, as well as different forms of condiments. The aim of this study was to measure the phytochemical content of raw spices (black cumin, garlic, ginger, and cardamom) and control hot red pepper (HRP), as well as the raw and cooked experimental and commercial spicy hot red pepper products. The samples were analyzed for phytochemical content using a spectrophotometer. Compared to raw experimental spicy hot red pepper, raw spices, and HRP, raw commercial spicy hot red pepper exhibited the highest bioactive phytochemicals. The cooked commercial spicy hot red pepper or sauté had the highest total flavonoid content (TFC) and total carotenoid content (TCC). Similarly, cooked experimental spicy hot red pepper contained the highest levels of total phenolic content (TPC) and β‐carotene. With *r* values ranging from 0.24 to 0.65, the TPC and TFC of raw spices were correlated with antioxidant activity. There was a significant correlation between TCC and 2,2‐diphenyl‐1‐picrylhydrazyl (DPPH) (*r* = .71), 2, 2′‐azino‐bis (3‐ethylbenzothiazoline‐6‐sulfonic acid) (ABTS) (*r* = .95), and ferric ion reducing antioxidant power (FRAP) (*r* = .76), as well as between β‐carotene and DPPH (*r* = .69), FRAP (*r* = .69), and ferrous ion chelating activity (FICA) (*r* = .78). This study verified that raw spices and their mix with hot red pepper are good sources of bioactive phytochemicals with radicals scavenging abilities in Ethiopian diets.

## Graphical abstract

Spices such as garlic, ginger, cardamom, and black cumin, as well as hot red pepper, and their mix were analyzed for phytochemical content. The analysis results showed that both raw and cooked products of spicy hot red pepper (Ethiopian berbere) improved phytochemical content. Mixing and cooking have an effect on the phytochemical content of spicy hot red pepper.

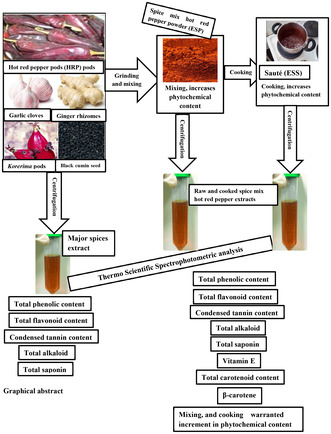

## 1 INTRODUCTION

The unique aromatizing, flavorful, antibacterial, preservative, and antioxidant properties of spices are well known for their appeal (Syamilah et al., 2022). Additionally, they are often utilized as food additives to enhance digestion because they are nutritionally significant elements (Derbie et al., 2018). According to Pop et al. (2019), common herbs and spices are generally recognized as safe (GRAS) for use as food additives. Herbs and spices bioactive components have therapeutic, health‐promoting, or disease‐ prevention effects in addition to being utilized to enhance their organoleptic qualities (Guldiken et al., 2018; Shahidi & Hossain, 2018). Contrarily, depending on the consumption dose, bioactive compounds found in herbs and spices may change and reverse into some hazardous properties (such as carcinogenic, genotoxic, neurotoxic, cytotoxic, teratogenic, hepatotoxic, and nephrotoxic effects) (Almotayri et al., 2020; Guldiken et al., 2018; Shahidi & Hossain, 2018). The colorful sections of herbs, vegetables, fruits, nuts, legumes, whole grains, and seeds are highly concentrated sources of phytochemicals, which are naturally occurring, non‐nutritive secondary metabolites of plant‐based bioactive compounds (Nigussie and Zemede, 2020). Due to their antioxidant and preservation properties, they may have positive effects on human health (Yusuf et al., 2018).

Consuming phytochemicals as part of a healthy diet can have positive effects on one's health in the form of antibacterial, anticancer, anti‐inflammatory, antiviral, and high antioxidant activity (Embuscado, 2019; Guldiken et al., 2018). Carotenoids, phenolic compounds, phytosterols, organosulfur compounds, phytoestrogens, glucosinolates and their degraded products, and dietary fiber are just a few of the many phytochemicals that can be found in functional foods and dietary supplements (Syamilah et al., 2022), in addition to terpenoids, vitamins, minerals, and nitrogen‐containing compounds (Embuscado, 2019). They significantly contribute to plant growth or protection against competitors, pathogens, or predators (Almotayri et al., 2020).

The most economically significant pepper species, *C. annuum*, is among the commonly domesticated pepper species (such as *C. baccatum*, *C. chinense*, *C. pubescen*, *C. frutescens*, and *C. annuum*), and is the focus of the study. It is a high‐value crop that is sold on the domestic and international markets and is consumed by households in large quantities in Ethiopia (Hailu et al., 2015). Although pepper offers only 25 Kcal per 100 g of energy, it is a great source of phytonutrients like β‐carotene (provitamin A), vitamins A, E, K, B2, and C, as well as minerals. According to (Kennedy & Wightman, 2011), it contains a variety of phytochemicals, including phenolic compounds, alkaloids, saponins, and terpenes. However, the variety, ripening stage, and growing conditions all affect the phytochemical content (Hernández‐Pérez et al., 2020). Ethiopian adults consume hot red pepper in fresh, dried, or processed forms (stew, hot sauté, paste), which is more than they consume of tomatoes and the majority of other vegetables (Gobie, 2019). For the production of hot red pepper powder, spices and herbs like cardamom (*Aframomum corrorima* (Braun) C.M. Jansen), ginger (*Zingiber officinale* Roscoe), garlic (*Allium sativum* L.), and black cumin (*Nigella sativa* L.) are commonly grown and used in Ethiopia (Gobie, 2019). They are also very important as functional foods and are rich sources of essential vitamins and minerals (Talía et al., 2020). They include a variety of other phytochemical substances as well. In order to maintain optimum health, these substances are known to prevent inflammatory illnesses linked to oxidative damage (Nazhand et al., 2020).

Since ancient time, food processing has been used to maintain and enhance the nutritional content, sensory qualities, and edibility of food, as well as release natural phytochemicals with functional and bioactive properties (Zhao et al., 2019). According to Raj and Arulmozhi (2013), spices are frequently consumed after cooking since they enhance the flavor, pungency, color, and deodorization of food and beverages. It is commonly perceived that cooked foods have a lower nutritional value than fresh foods and also have some unfavorable effects, such as the loss of nutrients (vitamin C), specific physicochemical characteristics, and the formation of toxic compounds with detrimental effects on organoleptic properties (Hwang et al., 2012). It is well known that heat treatment renders several phytochemical substances inactive. However, certain processing steps may enhance phytochemical status due to the transformation of phytochemicals into more active compounds, such as the deglycosylation of onion quercetin. Cooking causes compositional changes in peppers, such as capsaicinoid compounds (Hwang et al., 2012).

Ethiopian spicy hot red pepper powder, locally known as *berbere*, is made with at least 12 different herbs and spices, with occasional variations depending on the location. The mixing proportions vary from area to area, from house to house, and from processing enterprise to enterprise. Among the mixed recipes, spices such as black cumin, garlic, ginger, and Ethiopian cardamom were chosen as the subject of this study based on their amount used for spicy hot red pepper powder production. Furthermore, because of the presence of certain antinutritional components, the spices were taken into consideration for the study. Based on the aforementioned information, the current study examined the phytochemical composition of raw spices and hot red pepper (control) and raw and cooked experimental and commercial spicy hot red pepper products.

## 2 MATERIALS AND METHODS

### 2.1 Sample preparation

Fresh garlic cloves, ginger rhizomes, cardamom pods, dry hot red pepper pods, and black cumin seeds were cleaned, sun dried, and then separately finely ground in a laboratory setting after being collected from purposively selected spices processing enterprises. According to the procedure outlined by Medalcho et al. (2023), each of the non‐mixed raw spice powders (HRP, garlic, ginger, cardamom, and black cumin) as well as their proportionate mixture powder were developed from HRP (67.5%), garlic (13.5%), ginger (9.5%), cardamom (6.8%), and black cumin (2.7%), which was referred to as experimental spicy hot red pepper powder. Moreover, commercial spicy hot red pepper powder was collected from the same enterprises. It contained some additional herbs and spices in unknown proportions, and was the subject of this study. A portion of experimental and commercial spicy hot red pepper powders were cooked with water at 85°C for 7 min before being oven‐dried, finely ground, and sieved with 500 micrometer. Entire, finely ground samples were carefully packaged and stored until they were extracted.

### 2.2 Analytical procedures

#### 2.2.1 Phenolic compounds

##### Sample extraction

Following the homogenization of 1 g of each ground spice in 20 mL of ethanol:water (70:30, v/v), the aqueous ethanol extracts of the individual spices and the hot red pepper spice combination were created. The mixture underwent 30 s of vortexing and sonication. After that, Whatman no. 1 filter paper was used to filter the solution. The solvent of the mixed extracts was evaporated using a rotary vacuum evaporator at 50°C (Büchi Rotavapor R‐200, Finland) under reduced pressure. After that, the dried extracts were kept at 4°C in a dark room until further research (Ali et al., 2021).

##### Total phenolic content

The Folin–Ciocalteau method was used to calculate the quantity of TPC in each sample with certain adjustments and using gallic acid as a reference (Ali et al., 2021; Guldiken et al., 2018). Briefly, ten times diluted 1 mL of 10% Folin–Ciocalteu's reagent (v/v) (Sigma‐Aldrich® Merck KGaA, Darmstadt, Germany) was used to oxidize 0.1 mL of the extract (1 mg/mL). After waiting for 5 min, the mixture was neutralized with two milliliters (75 g/L) of 7.5% sodium carbonate. The plate was incubated for 40 min at 45°C. Finally, the absorbance of the resulting blue color was measured at 765 nm with the Thermo Scientific Multiscan Go Spectrophotometer (Thermo Fisher Scientific Oy Ratastie, Finland). The TPC was expressed as milligrams of gallic acid equivalent per gram of dry extract (mg GAE g^−1^) using the gallic acid calibration curve (*y* = 0.01811*x* + 0.2391, *R* ^2^ = .9973), and the findings were computed using the following equation:

$$C=\frac{c\times V}{m}$$

Where; *C* is the amount of total phenolics in dried extract given as milligrams of gallic acid equivalent per gram (mg GAE g^−1^), *c* is the concentration established from the gallic acid calibration curve (μg/mL), *V* is volume of extract solution in milliliter, and *m* is weight of dried extract in grams.

##### Total flavonoid content

The AlCl_3_ colorimetric method of Ali et al. (2021) was used to calculate the TFC with a few minor adjustments. The flavonoid‐aluminum complex's development into a yellow color served as the basis for the analysis. A 0.5 mL extract was mixed with 0.5 mL of methanol, 50 μL of 10% AlCl_3_, 50 μL of 1 mol L^−1^ potassium acetate, and 1.4 mL of water. The solution was incubated at room temperature for 30 min. The absorbance of the reaction mixture was measured at 415 nm in comparison to an ethanol blank. The TFC was estimated using a standard calibration curve of quercetin at 1–40 μg/mL (*y* = 0.2066*x* + 0.138, *R* ^2^ = .9812), and values were expressed as milligrams of quercetin equivalent per gram of dried extract (mg QE g^−1^) using the formula:

$$C=\frac{c\times V}{m}$$

Where; *C* stands for total flavonoid content expressed in milligrams of quercetin equivalent per gram of dried extract (mg QE g^−1^), *c* is the concentration established from the quercetin calibration curve (μg/mL), *V* stands for volume of extract solution in milliliter, and m is weight of dried extract in grams.

#### 2.2.2 Phyto‐antinutrients

##### Condensed tannin content

The vanillin condensation method described by Udu‐Ibiam et al. (2014) was used to evaluate the condensed tannin content (CTC) in an acid medium with little modification. 100 mg of each sample were extracted in 10 mL of 1% HCl in methanol (v/v). A mechanical shaker was used to shake the mixture for 24 h at room temperature. The mixture was filtered using Whatman no. 1 filter paper. The filtrate (1 mL) was mixed with 5 mL of vanillin HCl reagent that had been dissolved in methanol (4%, w/v) before being incubated at room temperature for 20 min in the dark. The absorbance was measured at 500 nm using a spectrophotometer. An aqueous solution of catechin (100–600 μg/mL) was used to create the calibration curve (*y* = 1.6141*x* + 0.2155, *R* ^2^ = .9821). Milligrams of catechin equivalent per gram of dry extract (mg CE g^−1^) were used to describe the outcomes.

##### Total alkaloids

A precise 5 g of each sample was weighed out and dissolved in 50 mL of a 10% acetic acid solution in ethanol. Before filtering, the mixture was thoroughly mixed and left to stand for 4 h. The filtrate was evaporated to one quarter (¼) of its original volume. Then, to precipitate the alkaloid, concentrated NH_4_OH was added drop by drop. The precipitate was removed with a weighted filter paper. The precipitate in the filter paper was weighed again after being stored in a desiccator and dried in an oven at 60°C for 30 min. The weight difference was typically used to calculate the weight of the whole alkaloid (Velavan, 2015). Then, the percentage of total alkaloids was calculated as follows:

$$\text{Totlaalkaloid}\left(\%\right)=\left(\frac{W2-W1}{W}\right)\times 100$$

Where; *W* is the sample weight, *W*1 is the empty filter paper's weight, and *W*2 is the weight of filter paper plus precipitate.

##### Total saponins

The technique of Udu‐Ibiam et al. (2014) was employed for total saponin analysis with a slight modification. A conical flask containing 20 g of each sample was filled with 100 mL of 20% aqueous ethanol. The samples were heated for 4 h at roughly 55°C with constant stirring (the solvent was evaporated until 40 mL of extract was produced), and after that, the mixture was filtered. 20 mL of diethyl ether was added to the residue in a 250 mL separating funnel, which was then vigorously shaken. The aqueous layer was recovered while the ether layer was cast off. After repeating the purification procedure, 60 mL of n‐butanol was added. The mixed n‐butanol extracts were rinsed twice in 10 mL of a 5% sodium chloride aqueous solution. The residual solution was heated in a water bath at around 90°C for 30 min. Following evaporation, the samples were dried in an oven to a consistent weight, stored in a desiccator, and then weighed again. The total saponin was calculated using equation 4, and the results were recorded.

$$\text{Total saponin}\;\left(\%\right)=\left(\frac{W2–W1}{W}\right)\times 100$$

Where *W* stands for the sample weight, *W*1 is the empty evaporation dish's weight, *W*2 is the weight of the evaporation dish plus saponin extract.

#### 2.2.3 Phytonutrients of HRP and spicy hot red pepper products

##### Total carotenoid content

Total carotenoid content (TCC) extraction was performed using methods from Asnin and Park (2015), with a few minor adjustments. One gram of each sample was vigorously shaken for 10 min at room temperature with 25 mL of the hexane:acetone mixture (6:4). After 15 min of centrifugation at 1500 rpm, the mixtures were filtered using Whatman no. 1 filter paper. Another container was used to collect the filtrate. The absorbance of the filtrate was assessed at 450 nm in contrast to the blank sample. The calibration curve (*y* = 0.0654*x* + 0.0736, *R* ^2^ = .9917) was created using β–carotene (20–100 μg/mL). The TCC was expressed as milligrams of β‐carotene equivalent per gram of dried sample (mg β‐CE g^−1^).

##### β‐Carotene

The sample preparation and β‐carotene content determination were carried out in accordance with Asnin and Park (2015) with slight modifications. One gram of each finely powdered sample was transferred to a 50‐mL disposable centrifuge tube, to which 10 mL of hexane:acetone (6:4) solvent was added and mixed for 10 min at room temperature. The solution was filtered through Whatman no. 1 filter paper. The filtrate was measured for absorbance at 663, 645, 505, and 453 nm when hexane: acetone (6:4) was used as a blank. According to equation 5, the amount of β‐carotene was determined and expressed as μg/kg of sample.

$$\begin{array}{c} \beta -\text{carotene}=0.216\times \mathrm{Ab}663–1.22\times \mathrm{Ab}645–0.304\times \mathrm{Ab}505\\ \;+0.452\times \mathrm{Ab}453 \end{array}$$

##### Vitamin E

The amount of vitamin E in the control and spicy hot red pepper samples was determined by a spectrophotometer according to the methods described by Asnin and Park (2015), with some modifications. One gram of each sample was extracted using a 10 mL mixture of absolute ethanol and 5% potassium hydroxide (10:1) and boiled for 30 min under reflux before adding 1 mL of petroleum ether. The extract mix was evaporated to dryness on a rotary evaporator. The residue was redissolved in a small amount in 1 mL of the proper carrier solvent (acetone). The absorbance of vitamin E was measured at 295 nm. The vitamin E standard calibration curve was created in the α‐tocopherol solvents at 1, 5, 10, and 15 μg/mL concentrations. Vitamin E was expressed as milligrams of α‐tocopherol equivalent per gram of dried sample (mg α‐TEg^−1^) using the equation based on the α‐tocopherol calibration curve (*y* = 10.061*x*−3.8236, *R* ^2^ = .9975).

#### 2.2.4 Antioxidant activity

For a correlation study with phytochemicals, the antioxidant activity of raw spices, including hot red pepper, and both raw and cooked spicy hot red pepper products was investigated. The antioxidant activity assay methods such as DPPH (Ali et al., 2021; Sokamte et al., 2019), ABTS (Bunea et al., 2013), FRAP (Li et al., 2017), and FICA (Sudan et al., 2014) were used with slight modifications.

### 2.3 Statistical analysis

Every parameter was tested twice, and mean values were reported at the statistical significance level of *p* < .05, along with their respective standard deviations (SD). Tukey's honestly significant differences (HSD) multiple rank tests were utilized for the statistical analysis of the data using the SAS JMP_14 software (Cary, North Carolina, USA). The correlation between phytochemicals and antioxidant activity assays was analyzed in individual raw spices as well as in both raw and cooked spicy hot red pepper products.

## 3 RESULTS AND DISCUSSION

### 3.1 Phytochemical content of spices

The findings demonstrated that the spices used in Ethiopia to produce spicy hot red pepper powder contained beneficial phytochemicals like phenolics, flavonoids, tannins, alkaloids, and saponins in varied amounts (Table 1). The TPC of raw spices was ranked in descending order as garlic, black cumin, cardamom, and ginger. Black cumin displayed the maximum TFC, followed by ginger and garlic, while cardamom was the last with significant (*p* < .05) differences.

**TABLE 1:** Phytochemical content of spices.

Spices	TPC (mg GAE g^−1^ dw) ± SD	TFC (mg QE g^−1^ dw) ± SD	CTC (mg CE g^−1^ dw) ± SD	Total alkaloid (%) ± SD	Total saponin (%) ± SD
Ginger	13.13 ± 0.92^a^	12.73 ± 1.89^b^	1.10 ± 0.15^a^	3.45 ± 1.74^b^	1.07 ± 0.28^a^
Black cumin	12.86 ± 1.22^b^	12.92 ± 1.93^a^	1.10 ± 0.18^a^	5.32 ± 0.21^a^	1.10 ± 0.15^a^
Garlic	13.06 ± 1.91^a^	12.65 ± 1.86^c^	1.05 ± 0.29^a^	5.44 ± 1.82^a^	1.05 ± 0.13^a^
Cardamom	12.77 ± 1.01^b^	12.43 ± 1.59^d^	1.12 ± 0.41^a^	3.90 ± 1.58^b^	1.06 ± 0.31^a^

*Note*: Levels of mean values that are not connected in the column by the same letter differ significantly at *p* < .05. Least squares means differences are separated by Tukey's HSD test at *α* = 0.05. SD stands for the standard deviation. [corrections added on 4 June 2024, after the first online publication: incorrect values were corrected.]

The TPC achieved in the current finding in garlic was lower than the earlier work of Alide et al. (2020) (32.17). The TPC in ginger was greater, according to Kelthoum et al. (2022) (29.78 mg GAE g^−1^). On the other hand, Ali et al. (2021) (3.30 mg GAE g^−1^) and Dessalegn et al. (2022) (0.25 mg GAE g^−1^) disagreed with TPC in cardamom. However, TPC in black cumin exceeded the research finding of Ali et al. (2021) (4.02 mg GAE g^−1^). According to Hasna et al. (2021), the TFC in garlic was 0.35 mg QE g^−1^, which was lower than the current findings. Similarly, lower TFC was reported by Akeem et al. (2016) in ginger (0.0014 mg QE g^−1^) and by Dessalegn et al. (2022) in Ethiopian cardamom (0.19 mg QE g^−1^). The TFC in black cumin contradicted the result obtained by Yous et al. (2021) (8.34 mg QE g^−1^). Due to the group of phytochemicals (such as phenolic compounds), the main component of spicy hot red pepper has the ability to contribute as a functional food.

The CTC of garlic was lower than that of all other spices, with significant differences at *p* < .05. The total alkaloids of spices ranged from 3.45% to 5.44% with black cumin and garlic, and ginger and cardamom did not differ from each other. Similarly, the total saponin content of all spices had not varied. Akeem et al. (2016), Hasna et al. (2021), and Yusuf et al. (2018) reported lower CTC in garlic (0.0011, 0.07, and 0.008 mg CE g^−1^, respectively) than the present finding, while Kelthoum et al. (2022) reported greater CTC in ginger (6.78 mg CE g^−1^). The presence of tannin in the present study contradicted the previous work of Akinduro et al. (2017), who noted the absence of tannin in ginger. The CTC of black cumin was lower than that of the Akinduro et al. (2017) study report (7.2 mg CE g^−1^). The total alkaloid of garlic extract agreed with the study finding of Muhammad and Idris (2019) (4.8%); however, it was higher than the work of Akeem et al. (2016) (1.21%). According to the study findings presented by Jayaratne et al. (2020), Nigerian ginger had a higher percentage (6.0) of total alkaloids. In accordance with a previous work of Akeem et al. (2016) in a similar genus of cardamom (*Aframomum melequeta*) (2.48%), which was disagreed with current finding in cardamom (3.90%). The total alkaloid content of black cumin was higher than that found in the earlier study by Giri et al. (2018), who reported less than 1%. The amount of total saponin in garlic was consistent with Akeem et al. (2016) finding (1.32%), but not with Yusuf et al. (2018) result (9.57%). A lower figure was observed for Nigerian ginger (0.80%) (Eleazu et al., 2012); however, the study finding given by Akeem et al. (2016) in ginger had a total saponin three times (3.57%) higher than the current discovery. The total saponin of cardamom was lower than that of the genus related to *Aframomum melequeta* (3.09%) (Akeem et al., 2016). Abbas et al. (2013) reported that black cumin had a greater total saponin content (4.54%) than the current one. Therefore, it is preferable for human diets to have less antinutrients.

In general, the variations in phytochemicals across studies on similar plants may be due to geographical location of sample collection, environmental factors (drying method, climate), growing and harvesting conditions (Hasna et al., 2021; Kelthoum et al., 2022; Shahidi & Hossain, 2018), extraction methods (Yusuf et al., 2018), and analytical tools and methodologies (Mošovská et al., 2016).

### 3.2 Phytochemical content of HRP and spicy hot red pepper products

As shown in Table 2, eight distinct phytochemicals (phenolics, flavonoids, tannins, alkaloids, saponins, vitamin E, carotenoids, and β‐carotene) were identified for HRP, experimental and commercial spicy hot red pepper powders, and cooked powders. An increase in TPC in mg GAE g^−1^ was noticed at *p* < .05 for four powder extracts of HRP<ESP<CSP<CSS<ESS. In comparison to the others, the TFC of CSS had the highest value, while CSP, ESS, ESP, and HRP were arranged in descending order sequentially. In addition to being appreciated for its economic significance, pungency, color, and aroma, hot red pepper (*Capsicum annuum* L.) is a significant ingredient with health‐promoting attributes (Alvarez‐Parrilla et al., 2011). In line with earlier research by Alvarez‐Parrilla et al. (2011) and Kim et al. (2019), the current result demonstrated that hot red pepper includes a variety of phytochemicals. The phytochemicals like phenolics, ascorbic acid, flavonoids, capsaicinoids, carotenoids, and tocopherols provide many nutritional and health benefits in preventing the oxidation of biomolecules, cancer, heart disease, diabetes, and lower serum cholesterol level (Yang et al., 2012). Asnin and Park (2015) observed a higher TPC (29.1 mg GAE g^−1^) in hot red pepper than the present finding. Conversely, the results of the current study were better than those of Shahidi and Hossain (2018) (3.53). Compared to the current data, Shahidi and Hossain (2018) reported higher TFC (21.3 mg QE g^−1^).

**TABLE 2:** Phytochemical content of HRP and spicy hot red pepper products.

Treatments	TPC (mg GAE g^−1^ dw) ± SD	TFC (mg QE g^−1^ dw) ± SD	CTC (mg CE g^−1^ dw) ± SD	Total alkaloid (%) ± SD	Total saponin (%) ± SD	Vit. E (mg α‐TE g^−1^ dw) ± SD	TCC (mg β‐CE g^−1^ dw) ± SD	β‐Carotene (μg kg^−1^) ± SD
HRP	12.90 ± 1.86^c^	13.21 ± 1.92^e^	1.06 ± 0.11^a^	5.26 ± 1.09^c^	1.08 ± 0.19^a^	63.16 ± 0.97^a^	100.62 ± 0.88^c^	4.65 ± 2.13^c^
ESP	13.62 ± 1.19^b^	13.85 ± 1.75^d^	1.10 ± 0.39^a^	8.05 ± 1.01^a^	1.10 ± 0.15^a^	63.20 ± 1.00^a^	101.41 ± 1.08^b^	4.64 ± 1.77^c^
ESS	14.21 ± 1.82^a^	14.14 ± 1.81^c^	1.07 ± 0.27^a^	5.60 ± 1.68^bc^	1.08 ± 0.28^a^	63.21 ± 0.95^a^	101.08 ± 1.55^bc^	5.11 ± 1.54^a^
CSP	13.76 ± 0.99^ab^	14.62 ± 1.73^b^	1.13 ± 0.19^a^	6.70 ± 0.99^b^	1.12 ± 0.13^a^	63.24 ± 1.76^a^	102.45 ± 0.95^a^	4.64 ± 0.39^c^
CSS	13.96 ± 1.08^ab^	15.14 ± 1.92^a^	1.12 ± 0.22^a^	4.40 ± 1.87^c^	1.08 ± 0.29^a^	63.24 ± 0.89^a^	102.93 ± 1.08^a^	5.06 ± 0.99^b^

*Note*: Levels of mean values that are not connected in the column by the same letter differ significantly at *p* < .05. Least squares means differences are separated by Tukey's HSD test at *α* = 0.05. SD stands for the standard deviation. HRP stands for control hot red pepper powder, ESP for experimental spicy hot red pepper powder, ESS for experimental spicy hot red pepper cooked product (sauté) powder, CSP for commercial spicy hot red pepper powder, and CSS for commercial spicy hot red pepper cooked product (sauté) powder. [corrections added on 4 June 2024, after the first online publication: incorrect values were corrected.]

Commercial spicy hot red pepper (CSP) had the greatest CTC (1.13 mg CE g^−1^), although all of the samples were identical to one another. On the other hand, the total alkaloid ranged from 4.40% to 8.05%, with CSS having the lowest value and ESP having the highest value. The mean total saponin values across all samples revealed no difference at *p* < .05. Adefegha and Oboh (2013) reported inconsistent CTC with the current result. The report of Otunola et al. (2010) (13.44%) conflicted with the overall alkaloid content of HRP. The same author observed higher total saponin in hot red pepper (7.40%).

The pattern for vitamin E and total saponin was similar. Although the TCC of CSP and CSS revealed no difference between them, they statistically (*p* < .05) differed from HRP and ESP. In terms of β‐carotene content, ESS and CSS were significantly ( < .05) different, but HRP, ESP, and CSP showed no differences from one another. The previous research of Hernández‐Pérez et al. (2020) and Mendes et al. (2020) (47.84 mg TE g^−1^) partially supported the vitamin E content of HRP. However, Mendes et al. (2020) did note a lower TCC in HRP. In contrast to the current observation, the same author reported a conflicting result for β‐carotene (13.10 mg/g).

A mixture of spices has the potential to be more nutritious and effective as preservatives than either spice alone (Nazhand et al., 2020). The well‐known spice mixture ‘*berbere*’, or *shiro*, is a key ingredient in almost all of the everyday foods prepared by Ethiopians (Melkegna et al., 2017). The TPC in ESP and CSP powder extracts was lower than that of Loizzo et al.'s (2011) earlier study on commercial Ethiopian spice blend *berbere* (71.3 mg chlorogenic acid E g^−1^), which is composed of cardamom, cinnamon, cloves, coriander, cumin, fenugreek, nutmeg, black pepper, turmeric, cayenne pepper, paprika, ginger, chopped onions, and garlic. Sinh et al. (2021) discovered similar results in cox, ginger, and pepper (20.52 mg GAE g^−1^). According to Loizzo et al. (2011), the TFC in Ethiopian spice blend *berbere* (32.5 mg QE g^−1^) was twice as great as the present findings of ESP and CSP. However, the findings of Abbas et al. (2013) and Sinh et al. (2021) were lower (1.79 mg QE g^−1^) than the current finding.

The experimental analysis result of CTC in ESP and CSP was lower than that of previous studies by Abbas et al. (2013) (2.82 mg tannic acid g^−1^) and Sinh et al. (2021) (3.2 mg tannic acid g^−1^). On the other hand, the earlier work by Loizzo et al. (2011) largely validated the total alkaloid in ESP (10.4%), but CSP was lower. Similarly, Joel et al. (2020) also noted a lower alkaloid content (0.51%) in a spice mix that included ginger, uziza (*Piper guineense*), uda (*Xylopia aethiopica*), and ehuru (*Monodora myristica*). The amount of total saponin in ESP and CSP disagreed with the report of the above‐mentioned author (0.25%).

The amount of vitamin E in ESP and CSP differed from the finding published by Ndife et al. (2020) in spice‐mix seasonings of *Utazi*, *Uziza*, *Nchuanwu*, *Uda*, groundnut, maggi, salt, ginger, and pepper (0.0014 mg α‐TE g^−1^). In comparison to the results of Hwang et al. (2012) for raw hot red pepper (1394.08 μg β‐CE g^−1^), the TCC in ESP and CSP was lower. According to the same author report, β‐carotene did not correspond with the results of ESP and CSP. The increased levels of vitamin E, TCC, and β‐carotene found in ESP and CSP may be attributable to the higher mixing fraction of hot red pepper in both spice combinations, as supported by Marianne et al. (2018). Hot red peppers are therefore employed as functional food additives, either on their own or in combination (Baenas et al., 2019).

In comparison to HRP, the levels of TPC, TFC, total alkaloid, and TCC significantly increased in the experimental spicy hot red pepper. This might be attributed to the garlic and ginger mixtures, which included more TPC, TFC, total alkaloids, and TCC than the other spices. The TFC, total alkaloid, and TCC of CSP were significantly higher than ESP; however, TPC, CTC, total saponin, vitamin E, and β‐carotene did not show any difference. This could be because of variations in the types and amounts of the main spices used, including fenugreek (*Trigonella foenum‐graecum*), cinnamon (*Cinnamomum verum*), rue (*Ruta chalepensis*), rosemary (*Rosmarinus officinalis*), koseret (*Lippia adoensis Var. Adoensis*), basil (*Ocimum basilicum*), coriander (*Coriandrum sativum*), onion (*Allium cepa*), white cumin (*Nigella sativa L*.), thyme (*Thymus vulgaris*), and clove (*Syzygium aromaticum)*.

This experimental results showed that TPC increased after cooking in ESS and CSS by 4.15% and 1.43%, respectively, and a similar pattern was observed for TFC in ESS and CSS, respectively, by 2.05% and 3.43%, which was confirmed by the finding of Hwang et al. (2012). The TPC in ESS and CSS was higher than the previous work of Sayin and Arslan (2015) in hot red pepper paste (mix of hot red pepper, spices, and herbs) in Turkish diet (1.7 mg/g) and Adegoke et al. (2016) in the formulated instant pepper soup mix (black pepper, garlic, negro pepper, calabash nutmeg, peppercorn, scent leaf, coriander, ginger, thyme, onions, and hot pepper) (8.89 mg GAE g^−1^). The TFC in the prepared pepper soup mix (6.35 mg QE g^−1^) (Adegoke et al., 2016) was lower than the values of ESS and CSS. These may be related to the breakdown of tough cell walls and the release of soluble flavonoids and phenolics from insoluble ester bonds, which improves the extractability of leaching bioactive compounds into the water, which is true if the leached bioactive compounds are taken into consideration or decanting is not used as a method of cooking (Alide et al., 2020). However, the inconsistent results reported by Zhao et al. (2019) may be the consequence of water‐soluble phenols that leached into the cooking water that was decanted (Nixon and Brian, 2020).

The CTC in ESS and CSS was partially consistent with the result of Adegoke et al. (2016) (0.94 mg CE g^−1^) in the formulated pepper soup mix. The same author reported that the identical product's total alkaloid content (8.89%) was higher than what was discovered in ESS and CSS. Total saponin in ESS and CSS showed a modest decrease when compared to raw ESP and CSP. The amount of total saponin lost during cooking for ESS was 11.82%, which contrasted with findings by Chaturvedi et al. (2012) (21.9%). Boiling in hot water decreases the antinutrients in plant products. But the later author report concurred on the loss of CSS (15.57%). This decrease during boiling may be caused by antinutrients leaking into the cooking water and being eliminated throughout the drying process (Nixon and Brian, 2020). Spices should be well cleaned and boiled to reduce antinutrient levels before consumption. However, spices make up a minor portion of the average person's diet.

The vitamin E content in cooked ESS and CSS agreed with Mendes et al. (2020) research findings that cooking did not affect the amount of α‐tocopherol in red pepper. Similar to this, Kuppithayanant (2014) noted that vitamin E is insoluble in water and comparatively resistant to heat. Both Nixon and Brian (2020) discovered a 13.5% loss of TCC in hot peppers during boiling, which was in contrast to the 0.33 percent loss in ESS. Contrarily, according to a number of researchers (Cai‐Hua et al., 2017; Hwang et al., 2012; Nixon and Brian, 2020), home cooking enhanced TCC in comparison to uncooked samples. This was in accordance with the percent raise in CSS (0.47). Adegoke et al. (2016) in formulated pepper soup mix reported TCC (880 μg β‐CE g^−1^) lower than in ESS and CSS. This phenomenon can be explained by cell wall disintegration and enhanced carotenoid extractability from plant materials containing carotenoids (Zhao et al., 2019). Additionally, cooking methods, the kind of food, and the composition of the food matrix may all have an impact on the thermal lability of carotenoids (Hwang et al., 2012; Nixon and Brian, 2020). The rate of β‐carotene change was inconsistent with findings of Cai‐Hua et al. (2017) and present result in ESS (9.2%) and CSS (8.30%).

Different cooking factors, such as duration, temperature, material‐to‐water ratio, food matrix, cooking method/condition, and the chemical make‐up of the phenolic component (that leaches into cooking water and decanting), might all contribute to the inconsistent results (Asogwa et al., 2021). However, the current investigation took into account the possibility that the raise in β‐carotene was brought on by leached β‐carotene (Alide et al., 2020). Cooking with leached bioactive chemicals improved food ingredient functional property. In general, TFC, TCC, and β‐carotene of CSS were substantially greater than ESS, although TPC, CTC, total alkaloid, total saponin, and vitamin E of ESS were not statistically different.

Generally, the same factor that was previously mentioned for the spices may also account for the variations in phytochemicals found in spicy hot red pepper or spice mixtures. Further contributing factors include mixed varieties, composition, proportion, and processing methods (Joel et al., 2020; Kelthoum et al., 2022; Nixon and Brian, 2020).

### 3.3 Correlation analysis

The antioxidant activities (DPPH, ABTS^+^, FRAP, and FICA) of raw spices and hot red pepper powders with their mixture in both raw and cooked form were analyzed to study the correlation against phytochemicals. According to the study's findings, there are substantial differences between the raw powder forms of spices and HRP and both raw and cooked products of the mixture. However, the cooked mixture or sauté powder showed the greatest increase in antioxidant activity following the raw mixture powder. The antioxidant activity was ranked in ascending order as follows: CSS > CSP > ESS > ESP > spices including HRP. Spices are good sources of phytochemicals; moreover, both raw and cooked mixture products are better sources of phytochemicals like flavonoids, vitamin E, β‐carotene, and carotenoids, and antioxidants (data not shown).

The results of the correlation study revealed that the examined phenolic compounds had a moderate correlation (*p* < .1) with tests for antioxidant activities (DPPH, ABTS, FRAP, and FICA) in raw spices (Table 3). A weak correlation was observed between TPC and DPPH free radical scavenging activity (IC_50_) (*r* = −.35). However, the connection between TPC and FICA chelating activity was also moderate (*r* = .65). On the other hand, TFC only had a minor association with FRAP (*r* = .24) and FICA (*r* = .41) and had a strong correlation with ABTS (IC_50_) (*r* = −.61).

**TABLE 3:** Correlation between phytochemicals and antioxidant activities of spices. [corrections added on 4 June 2024, after the first online publication: incorrect values were corrected.]

Variable	By variable	Correlation (*r*)	Count (*n*)	Signif Prob
TPC (mg GAE/g dw)	DPPH (IC_50_)	−.35	8	0.0396
TFC (mg QE/g dw)	ABTS (IC_50_)	−.61	8	.0419
TFC (mg QE/g dw)	FRAP (mg TE/g dw)	.24	8	0.0267
TPC (mg GAE/g dw)	FICA (mg QE/g dw)	.65	8	0.0304
TFC (mg QE/g dw)	FICA (mg QE/g dw)	.41	8	0.0193

The results of the current study in spices on the IC_50_ of DPPH and ABTS were in contrast to those of Zhang et al. (2013), who found a strong correlation between the phenolic and flavonoid contents and the IC_50_ of DPPH. Similar findings were reported by Sokamte et al. (2019), which contradicted the current findings that the IC_50_ of DPPH and ABTS were closely linked with total phenolics (*r* = .93 and *r* = .89) and flavonoids (*r* = .94 and *r* = .92), respectively. The IC_50_ was negatively correlated, which means free radical scavenging activity was positively correlated with phenolics and flavonoids (Sokamte et al., 2019; Zhang et al., 2013). Several studies found a substantial correlation between FRAP and FICA antioxidant activities and phenolic content (Chou et al., 2021; Zhu et al., 2021), which was inconsistent with current findings (moderate).

In HRP and raw and cooked spicy hot red pepper products, the TCC and β‐carotene showed good correlation with antioxidant activities (DPPH, ABTS, FRAP, and FICA) at *p* < .05 (Table 4). However, phytochemicals that were not significantly correlated (*p* < .05) with antioxidant activities were excluded (data not shown). The TCC and ABTS had a strong association (*r* = .95), while FRAP and DPPH had moderate correlations (*r* = .76 and *r* = .71, respectively) at *p* < .05. The moderate correlation between β‐carotene and FICA, DPPH, and FRAP, in that order, *r* = .78, .69, and .69. Similar to the current findings in control and spicy hot red pepper, Bhandari et al. (2016) revealed a significant association between TCC and the antioxidant properties of foods, like between β‐carotene and antioxidant activity. However, we were unable to detect a correlation between TCC and FICA, β‐caroteneand ABTS activity at *p* < .05.

**TABLE 4:** Correlation between phytochemicals and antioxidant activities of HRP and spicy hot red pepper products.

Variable	By variable	Coefficient (*r*)	Count (*n*)	Signif Prob
Total carotenoids (mg BCE g^−1^)	DPPH (IC_50_)	.71	10	.0217
Total carotenoids (mg BCE g^−1^)	ABTS (IC_50_)	.95	10	<.0001
Total carotenoids (mg BCE g^−1^)	FRAP (mg TE g^−1^)	.76	10	.0100
β‐carotene (ppm)	DPPH (IC_50_)	.69	10	.0261
β‐carotene (ppm)	FRAP (mg TE g^−1^)	.69	10	.0272
β‐carotene (ppm)	FICA (mg QE g^−1^)	.78	10	.0075

In contrast to the current findings, a substantial association between TPC and antioxidant activities was observed by Tangkanakul et al. (2012) in Thai sauces. The variety of examined samples, various antioxidant testing techniques, concentrations, and particular sample circumstances are some of the variables that affect the correlation. Additionally, the composition and any other chemicals found in the extract have a synergistic, antagonistic, or additive impact (Niciforovic et al., 2010).

## 4 CONCLUSIONS AND RECOMMENDATION

Ethiopian cuisine relies heavily on spices to improve its flavor, appearance, color, aroma, pungency, and palatability. The raw spices (black cumin, ginger, cardamom, garlic, and HRP) and spicy hot red pepper powders in Ethiopia have bioactive compounds that serve as sources of nutrients and enhance significant functional properties when consumed. The study results demonstrated a substantial raise in TPC, TFC, total alkaloid, and TCC when raw spices and hot red pepper powders were mixed. The TPC, TFC, total alkaloid, and β‐carotene of spice mix powder were also markedly increased by cooking. However, a negligible decrease in CTC, total saponin, and TCC was seen in the cooked product, while vitamin E was maintained. Spices mixed during spicy hot red pepper powder production are thus prospective sources of phytochemicals. As a result, combining spices and cooking the mixture enhances their health‐promoting antioxidant qualities. However, only four basic spices were selected for this investigation among the at least 12 herbs and spices that are used to make Ethiopian spicy hot red pepper powder in a limited‐scope laboratory study. Therefore, additional research is recommended to determine the types and mixing ratio of spices used to produce spicy hot red pepper powder based on the practices of the communities and spices processing businesses, the effect of cooking time and temperature combination, and the bioactive phytochemicals in remaining herbs and spices with their functional properties.

## AUTHOR CONTRIBUTIONS

**Tadewos Hadero Medalcho:** Conceptualization (lead); data curation (lead); formal analysis (lead); funding acquisition (lead); investigation (lead); methodology (lead); project administration (lead); resources (equal); software (equal); supervision (equal); validation (equal); visualization (equal); writing – original draft (lead); writing – review and editing (equal). **Engeda Dessalegn Augchew:** Conceptualization (equal); methodology (equal); supervision (equal); validation (equal); visualization (equal); writing – review and editing (equal). **Kebede Abegaz Ali:** Conceptualization (equal); validation; supervision (equal). **Juan Ignacio Mate:** Conceptualization (equal); methodology (equal); resources (equal); supervision (equal); validation (equal); visualization (equal).

## FUNDING INFORMATION

This research did not receive any specific grants from funding agencies in the public, commercial, or not‐for‐profit sectors.

## CONFLICT OF INTEREST STATEMENT

The authors declare that they have no conflicts of interest.

## ETHICS STATEMENT

This research did not contain any animal or human experiments.

## Data Availability

All relevant data for this article are included within the article.
